# Identification of the Yarrowia lipolytica cysteine sulfinic acid decarboxylase gene using a newly developed method with optimized Escherichia coli combinations of mutant alleles

**DOI:** 10.1099/mic.0.001620

**Published:** 2025-11-04

**Authors:** Masanobu Nishikawa

**Affiliations:** 1 Research Institute for Biological Sciences Okayama (RIBS Okayama), Okayama, Japan

**Keywords:** cysteine sulfinic acid decarboxylase, *Escherichia coli*, potassium-dependent small conductance mechanosensitive channel MscK, taurine biosynthesis, *Yarrowia lipolytica*

## Abstract

To develop a low-cost, environmentally friendly taurine fermentation method for sustainable marine fish culture using feed derived from photosynthetically produced agricultural products, it is crucial to study cysteine sulfinic acid decarboxylase (CSAD), a key enzyme in the taurine biosynthetic pathway in applicable microorganisms. In this study, a method was devised to screen for CSAD genes using *Escherichia coli* growth as an indicator, based on sulfur assimilation following the decarboxylation of l-cysteic acid, a taurine precursor compound. The *E. coli* used has a double deletion mutation of *cysA* (sulfate/thiosulfate ABC transporter) and *ssuD* (FMNH_2_-dependent alkanesulfonate monooxygenase) genes. If needed, an additional defect in enzyme genes, such as *cysC* (adenylyl-sulfate kinase), which participates in the pathway reducing sulfate to sulfite, is also introduced. Using this method, it was demonstrated that the glutamic acid decarboxylase gene from *Yarrowia lipolytica* possesses CSAD activity. The identified decarboxylase was further confirmed to act on l-cysteine sulfinic acid. Additionally, two observations made during method refinement to reduce background growth in screening are discussed: that SsuD is involved in sulfur assimilation from an unknown sulfur compound and that certain *mscK* (mechanosensitive channel) missense mutations enable external sulfate above a specific concentration to enter the cell.

## Data Summary

The complete nucleotide sequence of pTA-YlC1 has been registered in DDBJ (LC858821).

## Introduction

Taurine exhibits various physiological activities [1,4]. It is an essential nutrient for fish growth, but marine fish, especially juveniles, have low biosynthetic capacity for taurine and obtain it from prey [5,9]. Recently, concerns over the depletion of small fish resources like sardines used in marine fish farming have shifted attention to the sustainability of plant-derived feed sourced from photosynthesis. However, reports of taurine content in plant-derived agricultural products are extremely rare [10], and little attention has been paid to its presence. As a result, chemically synthesized taurine must currently be added to plant-based feed. To enhance sustainability and meet growing demand, developing microbial fermentation technology for taurine is crucial. Microorganisms for this purpose must be nontoxic to both the cultivated fish and human consumers. Suitable candidates include ‘Generally Recognized as Safe’ species like *Yarrowia lipolytica* known as ‘oleaginous yeast’ and *Bacillus subtilis* variant natto used in the production of natto (fermented soybeans). Taurine biosynthesis by *Y. lipolytica* has been reported [11]. While not a natto producer, taurine has also been detected in soy milk fermented by the related bacterium *B. subtilis* [12]. Several pathways for taurine biosynthesis exist, including one via l-cysteine in organisms like mammals, invertebrates, algae, marine cyanobacteria and marine flavobacteria [13,15].

As shown in Fig. S1 (available in the online Supplementary Material), l-cysteine is converted to l-cysteine sulfinic acid by cysteine dioxygenase (CDO), which is then converted to hypotaurine by cysteine sulfinic acid decarboxylase (CSAD) and finally oxidized to taurine. Another potential pathway involves l-cysteine sulfinic acid being oxidized to l-cysteic acid, which is then decarboxylated to taurine by the same CSAD. With advancements in genome sequencing, candidate genes annotated as CDO and CSAD have been identified in *Y. lipolytica* yeast and *B. subtilis* var. *natto*, but their authenticity remains unconfirmed.

In this study, to identify CSAD genes essential for advancing research and development in taurine fermentation, I aimed to develop a novel method inspired by sulfur assimilation in *Escherichia coli* carrying specific combinations of mutations as an indicator (Fig. S2). *E. coli* can utilize l-cysteic acid or taurine as sole sulfur sources [16,18]. Sulfur assimilation from l-cysteic acid in *E. coli* depends on two-component alkanesulfonate monooxygenase system (SsuDE) [17], whereas assimilation from taurine requires taurine dioxygenase (TauD) or SsuDE [18,21]. However, it has not been fully verified whether underivatized taurine serves as a direct substrate for SsuD. By deleting *ssuD*, it should be possible to engineer an artificial organism unable to assimilate sulfur from l-cysteic acid but capable of assimilating sulfur from taurine.

As demonstrated in our previous reports [20], in the presence of 0.5 mM taurine and trace amounts of sulfate in the culture medium, a *tauD*-deficient *E. coli* strain with *ΔcysN* (sulfate adenylyltransferase subunit 1) cannot immediately assimilate sulfur from taurine and consequently fails to grow. However, with extended culture periods, mutant strains capable of assimilating sulfur from taurine emerge and are selected for growth. This growth relies on SsuDE-dependent desulfonation of taurine. The inactivation of taurine sulfur assimilation, which might seem unusual when alternative sulfur sources are essential, is linked to the function of the sulfate transporter CysUWA/CysP/Sbp. The mechanism involves trace extracellular sulfate entering cells via the CysUWA transporter with Sbp, suppressing the expression of *ssuEADCB* and *tauABCD*. Over time, dysfunctional mutants of the transporter genes are selected, allowing SsuDE-dependent sulfur assimilation to commence. When designing a screening system reliant on TauD function, it is crucial to consider the inhibitory effects of trace sulfate influx on *tauD* gene expression.

The principle of identifying the *CSAD* gene is to observe its functional expression in *E. coli* cells. Specifically, I aimed to detect the emergence of recombinant clones that had acquired genes enabling sulfur assimilation via the decarboxylation of l-cysteic acid to taurine under conditions where sulfur assimilation from alternative sources, such as sulfate, was suppressed. The screening method for identifying CSAD at the conceptual stage involves the following steps (Fig. S2). SsuD is removed from *E. coli* to shift sulfur assimilation from l-cysteic acid to the TauD-dependent pathway, which cleaves the C–S bond in taurine through oxygenolytic desulfonation. To eliminate sulfate from cells, which is a readily available sulfur source that can inhibit *tauD* expression, CysA, a component of the sole sulfate ion transporter, is removed. Since *E. coli* does not naturally possess CSAD, which converts l-cysteic acid to taurine, introducing an exogenous CSAD gene should allow the engineered *E. coli* to use l-cysteic acid as a sulfur source via the TauD pathway. Through rigorous experimentation, I successfully identified the *Y. lipolytica* CSAD gene using the newly developed method. Additionally, while exploring strategies to reduce false positives in CSAD screening, I discovered that SsuD is involved in sulfur assimilation from an unknown sulfur compound and that the *mscK* (potassium dependent, small conductance mechanosensitive channel) mutation surprisingly contributes to sulfate influx, both of which are reported here.

## Methods

### Bacterial strains, cultivation media and cultivation condition

The *E. coli* K12-derived strains used in this study are listed in Table 1. Mutants lacking *cysA* were selected based on chromate resistance [22,23], while *lacI* (DNA-binding transcriptional repressor) mutants that constitutively promote expression from the *lac* promoter were selected based on phenyl-*β*-d-galactoside resistance [24]. Although the *lacI* feature was not directly utilized in this study, it is mentioned to clarify the genetic background of CR2 and its derivatives. To assess sulfur utilization, a small number of cells from a fresh overnight culture of *E. coli* strains grown on Miller’s ‘lysogeny broth’ or ‘Luria-Bertani’ (LB) agar medium [25] were streaked onto minimal agar plates and incubated at 37 °C for 3 to 4 days. Davis minimal agar medium, purchased from Difco Laboratories, consisted of 1 g d-glucose, 7 g dipotassium hydrogen phosphate, 2 g potassium dihydrogen phosphate, 0.5 g sodium citrate, 0.1 g magnesium sulfate, 1 g ammonium sulfate and 15 g agar in 1 l. Although the manufacturer’s documentation does not explicitly mention it, it is assumed that the sodium citrate and magnesium sulfate are in the forms of trisodium salt dihydrate and heptahydrate, respectively. Modified M9 minimal agar medium [18] was prepared using 2 g d-glucose, 8.5 g disodium hydrogen phosphate dihydrate, 3 g potassium dihydrogen phosphate, 0.5 g sodium chloride, 1 g ammonium chloride, 11 mg calcium chloride, 203 mg magnesium chloride hexahydrate and 15 g agar in 1 l. Depending on the experimental requirements, ammonium sulfate and other substances were added to the modified M9 medium (see Fig. S3 legend for details).

**Table 1.** *E. coli* strains and plasmids used in this study

Strain or plasmid	Description	Source or reference
MG1655 (Seq)	*F^-^ λ^-^ rph−1*	CGSC7740
CR2	MG1655 *ΔcysA* (Ala146_Arg149del) *lacI1* (T332A, Val111Glu)	This study
CR2S	CR2 *ΔssuD751*::kan	This study
CR2S-M1	CR2S *mscK1* (T2372C, Leu791Pro) *atoC1* (C413T, Thr138Ile) *ydhB1* (C476T, Ala159Val)	This study
CR2S-M2	CR2S *mscK2* (A2584T, Asn862Tyr)	This study
CR2S-M3	CR2S *mscK3* (G2594A, Gly865Asp)	This study
CR2S-M1-c	A clone with deleted *cysC* derived from CR2S-M1; *cysC*::Cm	This study
CR2S-M1-d	A clone with deleted *mscK* derived from CR2S-M1; *mscK*::Cm	This study
CR2S-M1-2	A clone with NaCl-resistant sulfate-sulfur assimilation derived from CR2S-M1; *ΔclsA* (Asn232_Asp244del) *mscS*^WT^	This study
CR2S-M1-3	A clone with NaCl-resistant sulfate-sulfur assimilation derived from CR2S-M1; *clsA*^WT^ *mscK1,-4* (T2372C, Leu791Pro; G392T, Arg131Leu)	This study
CR2SC	CR2S *cysC*::Cm	This study
R8	BW25113 *ΔcysN753*::frt *ΔssuD751*::frt *ΔtauD736*::frt	[18]
pTA	*oriC bla trpL*p (promoter element of *trpLEDCBA* operon)	This study
pTA-*Yl*C1	*oriC bla trpL*p-*CSAD* (chemically synthesized *Y. lipolytica* CSAD gene)	This study

### PCR detection of *mscK* allele

PCR was performed using genomic DNA extracted from each strain as a template, with a primer set comprising 5′-ATCCACGGTGACAGAAGAACGGCG-3′ and 5′-CCGGCATGAACAACGCGCACTTTG-3′.

### Plasmid construction

A potential CSAD candidate was identified through a blast search against the genome data of *Y. lipolytica*. The corresponding coding sequence (CDS) was chemically synthesized, and the plasmid containing the CDS under the control of the *E. coli trpL* promoter, an ampicillin resistance gene, and a replication origin was designated as pTA-*Yl*C1 (Table 1). An empty vector, pTA, was also prepared.

### Verification of CSAD activity using a resting cell reaction system

Two lines of *E. coli* strain CR2SC harbouring pTA-*Yl*C1 and pTA, respectively, were prepared for whole-cell biocatalysis. Cells were harvested from overnight cultures in fresh LB broth (40 ml) containing ampicillin (50 µg ml^−1^), washed twice with 50 mM potassium phosphate buffer (pH 7) and resuspended in 5 ml of the same buffer. An aqueous solution of l-cysteic acid was added to a 1.6 ml aliquot of the cell suspension to a final volume of 2 ml, achieving a final l-cysteic acid concentration of 2 mg ml^−1^. A similar experiment, without l-cysteic acid, was performed as a control. Tubes containing the resting cell reaction system were shaken overnight at 30 °C. After centrifugation to remove cells, the supernatants were either directly analysed by TLC or derivatized for GC-MS without any concentration.

### TLC

For TLC, 2 µl of each sample was spotted onto a silica gel 60 high-performance TLC plate (Supelco). The plate was developed using a solvent system of 1-butanol, acetic acid and water in a 3 : 1 : 1 ratio. Ninhydrin reagent was used to visualize amine-containing analytes.

### GC-MS

For GC-MS, 0.4 ml of each sample was derivatized using a sulfonic acid-specific method developed by Kataoka *et al*. [26], converting taurine to 2-(isobutoxycarbonylamino)-*N*,*N*-dibutylethanesulfonamide. The derivatized product was dissolved in 1 ml of ethyl acetate, and 1 µl of the solution was injected into a Clarus680-SQ8T GC-MS system (PerkinElmer) equipped with a low-polarity capillary column, DB-5ms (Agilent Technologies) (length, 30 m; inner diameter, 0.25 mm; and film thickness, 0.25 µm). GC-MS conditions were as follows: helium as the carrier gas; column flow rate, 1 ml min^−1^; injector temperature, 200 °C; split ratio, 1 : 50; oven temperature ramp, 80–260 °C (9 °C min^−1^), followed by a 10 min hold at 260 °C; and ion source temperature, 200 °C.

### Substrate specificity of recombinant CSAD enzyme

A small amount (240 µl) of *E. coli* strain R8 (*ΔcysN ΔssuD ΔtauD*) cells harbouring pTA-*Yl*C1, previously cultured in LB broth with ampicillin, was transferred to 120 ml of fresh LB broth of the same composition. After 2 h of incubation at 37 °C, 3-indoleacrylic acid was added to the culture medium (100 µg ml^−1^), followed by further incubation for 17 h. Cells were collected from 80 ml of the culture, washed with 50 mM potassium phosphate buffer (pH 7) containing 0.5 mM DTT and 0.5 mM EDTA and disrupted using intermittent ultrasonic waves. For the control experiment, R8 containing pTA was processed in the same manner. The crude cell-free extract, obtained as the supernatant after centrifugation, was introduced into the following enzyme reaction system buffered with 50 mM potassium phosphate (pH 7): l-cysteine sulfinic acid or l-cysteic acid, 50 mM; pyridoxal phosphate, 60 µM; DTT, 0.5 mM; EDTA, 0.5 mM; and total volume, 200 µl. After incubation at 37 °C for 3 h, the samples, accompanied by 10 μmoles of homotaurine (internal standard), were subjected to GC-MS analysis following chemical derivatization as described above.

## Results

### Combinations of mutations that should be present in *E. coli* for screening of CSAD

**Fig. 1.**
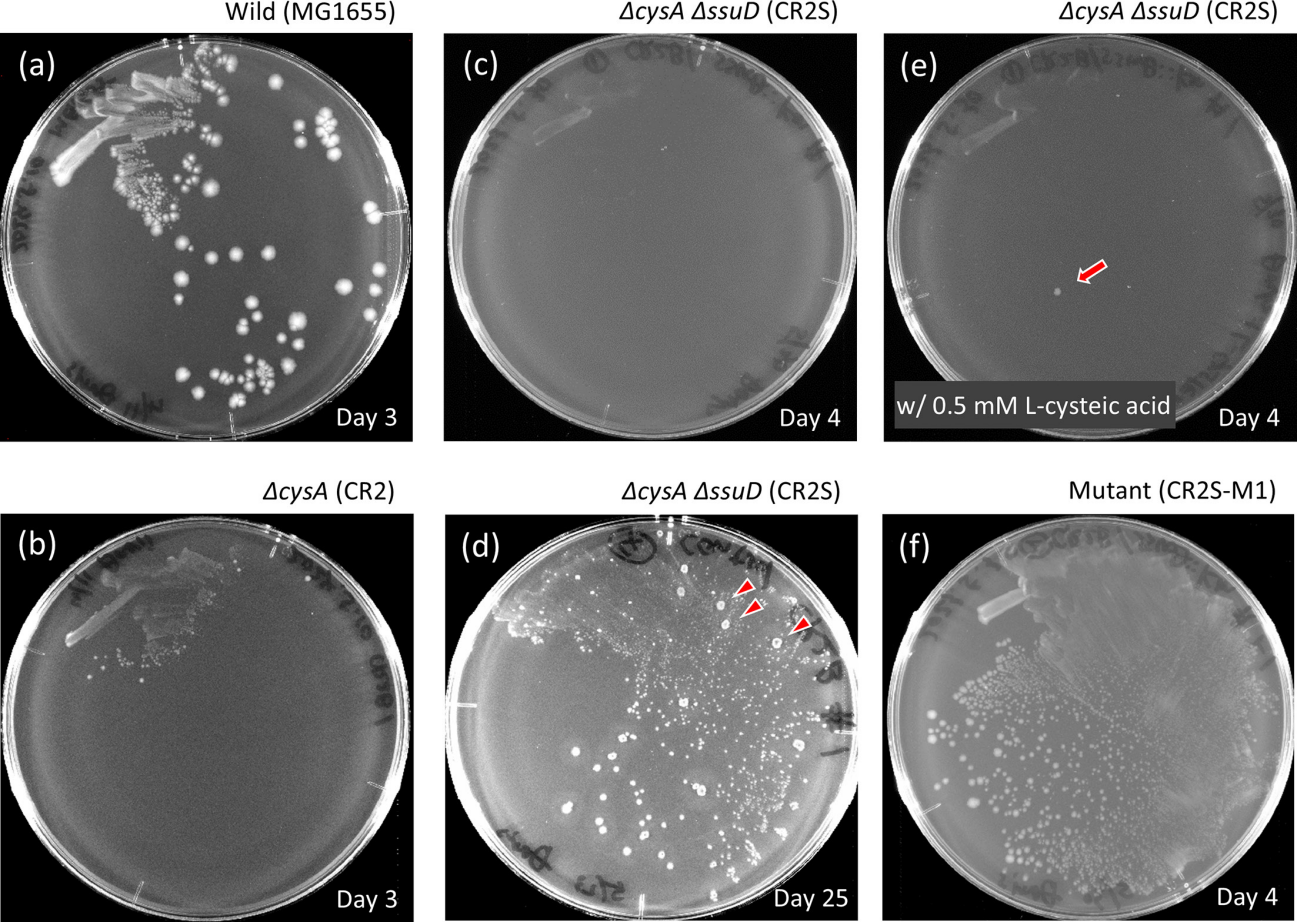
Suppression of undesired sulfur utilization during screening of the CSAD gene and emergence of resistant mutants. *E. coli* strains were streaked onto Davis minimal agar (a–d, f) or Davis minimal agar supplemented with 0.5 mM l-cysteic acid (e). The cells were incubated at 37 °C for periods ranging from 3 to 25 days, as indicated in each panel. Arrowheads and an arrow highlight colonies of putative mutants. A clone designated CR2S-M1 was isolated from the colony indicated by the arrow in panel (e) and subsequently streaked on LB agar for further analysis.

**Fig. 2.**
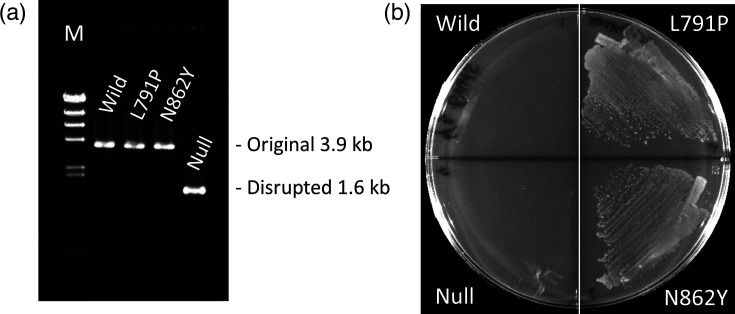
Identification of *mscK* missense mutations conferring ‘gain-of-function’ phenotypes, enabling sulfate uptake. (**a**) PCR analysis of *mscK* alleles. Genomic DNA from each strain was subjected to specific PCR amplification to detect DNA fragments covering the *mscK* allele. The resulting PCR products were analysed via agarose gel electrophoresis. (**b**) Sulfate-sulfur assimilation assay. Strains were streaked on Davis minimal agar and cultured at 37 °C for 3 days. Strains tested included wild (CR2S, *mscK*^WT^), L791P (CR2S-M1, *mscK1*), N862Y (CR2S-M2, *mscK2*) and null (CR2S-M1-d, *mscK*::Cm). The *mscK*::Cm allele replaces nearly the full-length CDS with a chloramphenicol resistance gene.

For culturing *E. coli*, commercially available premixed Davis minimal agar was used, despite its limitation that ingredient concentrations cannot be arbitrarily adjusted. The convenience of being ready to use after autoclaving was prioritized. This medium contains sulfate ions from ammonium and magnesium salts (8.4 mM) and sulfate derived from agar (estimated to be about 2.8 mM), either in free form or as sulfate esters. The WT parent strain MG1655 grew well by utilizing sulfur from sulfate (Fig. 1a). Based on experiments with M9 minimal medium, the CysUWA/Sbp/CysP system appears to be the only functional sulfate transporter [27]. However, the growth of strain CR2, which is expected to lack sulfate uptake due to its nonfunctional *cysA*, was not completely suppressed (Fig. 1b). Interestingly, strain CR2S, in which *ssuD* was disrupted to block l-cysteic acid sulfur assimilation, completely abolished the weak growth seen in CR2 (Fig. 1c). Thus, introducing a double deletion of *cysA* and *ssuD* into *E. coli* seemed to effectively eliminate background growth during CSAD gene screening by preventing sulfur assimilation from sulfate in the agar medium and unknown sulfonic acid compounds. However, when the incubation period was extended to 25 days, background growth re-emerged (Fig. 1d). Nearly all CR2S cells were observed to grow slowly using an unidentified sulfur source, and colonies exhibiting accelerated growth, suspected to be mutants, also appeared (Fig. 1d, arrowheads). As a result, the screening needed to be completed within 4 to 5 days. Next, when CR2S cells were cultured on Davis minimal agar supplemented with 0.5 mM l-cysteic acid, a substrate of CSAD, putative mutants appeared by the fourth day, even though the cells were not provided with a CSAD gene (Fig. 1e, arrow). A presumptive mutant obtained through single-colony isolation was designated CR2S-M1. This strain also grew well on Davis minimal agar lacking l-cysteic acid (Fig. 1f), suggesting that it can utilize an unknown sulfur source in the medium. l-cysteic acid in the medium appears to promote the emergence of mutants capable of utilizing unknown sulfur sources rather than directly supplying sulfur to cells. To identify the genes responsible for the phenotypes of CR2S-M1 that may hinder CSAD gene screening by producing false positives and to determine the sulfur source it utilizes, several candidate genes, including *tsuA* (thiosulfate transporter) [27], *yidJ* (putative sulfatase/phosphatase) [28] and *yihT* (6-deoxy-6-sulfofructose-1-phosphate aldolase) [29], were disrupted one by one in CR2S-M1. The growth of these disruptants on Davis minimal agar was examined while the whole genome of CR2S-M1 was sequenced. Among these, a disruptant CR2S-M1-c, in which *cysC* (adenylyl-sulfate kinase) was deleted from CR2S-M1, no longer grew, revealing that CR2S-M1 utilizes sulfur from sulfate. Since *cysA*, a component of the sulfate transporter believed to be the only one, was already disrupted, the discovery of another sulfate transport mechanism was unexpected. Whole genome analysis revealed that CR2S-M1 had a missense mutation in the *mscK* gene, designated as *mscK1* in this study. Other mutants derived from the same parent strain CR2S, namely, CR2S-M2 and CR2S-M3, obtained through the same isolation process as CR2S-M1, also grew on Davis minimal agar like CR2S-M1. These mutants had distinct missense mutations in the *mscK* gene, designated as *mscK2* and *mscK3*, respectively (Table 1). When the *mscK1* gene was deleted from the CR2S-M1 genome, the strain could no longer grow on Davis minimal agar, behaving similarly to the parent strain CR2S (Fig. 2). Therefore, *mscK1* represents a ‘gain-of-function’ mutation that is thought to enhance membrane permeability to sulfate.

To investigate how the *mscK* mutation compensates for the *cysA* deficiency in this study, the strain was cultured on modified M9 minimal agar, designed to mimic the composition of commercially available Davis minimal medium, for 3–7 days (Fig. S3). CR2S-M1 (*ΔcysA ΔssuD mscK1*) could not grow on modified M9 medium with a low sulfate concentration (~2.8 mM) (Fig. S3A). In contrast, the strain grew on modified M9 agar containing 11.2 mM sulfate (Fig. S3B). These findings suggest that sulfate influx through the *mscK1*-encoded channel [MscK1 (Leu791Pro)] occurs only at sulfate concentrations higher than those required for active transport through CysUWA/Sbp/CysP. The modified M9 medium used in this study significantly differs from the Davis medium, especially in sodium and potassium levels where the *mscK1* mutant was isolated. Consequently, the effect of these ion concentrations on the sulfate uptake by MscK1 was investigated. Sulfate assimilation via MscK1 was inhibited by 204 mM Na^+^ but not by 122 mM K^+^ (Fig. S3C–F). In a control experiment, replacing sulfate with taurine did not inhibit growth in the presence of 204 mM Na^+^ (Fig. S3G–H). This indicates that growth inhibition is due to the inhibition of sulfate influx by Na^+^, rather than direct cytotoxicity. When the incubation period was extended from 3 to 7 days in the presence of 204 mM Na^+^, several colonies suspected to be mutants were observed (Fig. S3D2 and E2). Two clones isolated individually from these colonies exhibited immediate growth on a fresh Davis minimal agar plate with 204 mM Na^+^ (data not shown), suggesting them to be mutants originating from CR2S-M1, and they were designated as CR2S-M1-2 and -3 (Table 1). The *mscK* gene in strain CR2S-M1-2 was confirmed to be identical to the *mscK1* allele of its progenitor strain, CR2S-M1. The *mscS* (small conductance mechanosensitive channel MscS) [30,31], structurally similar to *mscK*, remained a WT allele, indicating the presence of an unknown mutation in another gene. Whole-genome analysis of CR2S-M1-2 identified a novel mutation in *clsA* (cardiolipin synthase A). In the *mscK* gene of strain CR2S-M1-3, a new missense mutation (R131L) was found accompanying the previously identified missense mutation (L791P). However, further analysis of these mutants has yet to be conducted.

In conclusion, to suppress background growth in *E. coli* during CSAD screening, introducing mutations in *cysA* and *ssuD* is preferable. Additionally, deleting *cysC* and/or *mscK* as appropriate may further reduce background growth.

### Identification of *Y*. *lipolytica* CSAD gene

Taurine production in *Y. lipolytica* has been reported [11]. Although the report provides limited details, it is likely that CSAD plays a role in the process. From an enzymatic chemical perspective, CSAD is anticipated to be highly analogous to glutamic acid decarboxylase (GAD) [15]. According to the KEGG database for *Y. lipolytica* strain DSM3286 (KEGG:yli), two putative GAD genes (YALI2_B00475g and YALI2_F00165g) have been found [32]. Recently, enzymes encoded by two genes from another *Y. lipolytica* strain, CLIB122 (YALI0C16753g (*YlGAD2*) and YALI0F08415g (*YlGAD1*)), were confirmed to exhibit GAD activity [33]. However, it remains uncertain whether one or both of these GAD isozyme genes are responsible for CSAD activity, enabling l-cysteic acid sulfur assimilation. A blast search was conducted against the genome of *Y. lipolytica* CLIB122 [34] using the amino acid (aa) sequence of human CSAD (GenBank: AAH98342.1) [35] as a query. A protein annotated as ‘glutamate or tyrosine decarboxylase or a related PLP-dependent protein’ (GenBank: CAG82237.1, YALI0C16753p) was identified with the highest likelihood. A gene encoding an identical aa sequence is also present in *Y. lipolytica* CLIB89 (W29) (YALI1_C23902g) [36]. Strain DSM3286 has a variant gene (YALI2_B00475g) with two aa substitutions (S181G and E344D). Hébert *et al*. suggested that YALI0C16753p might function as a CSAD [11], but their findings did not definitively conclude that it is a CSAD.

**Fig. 3.**
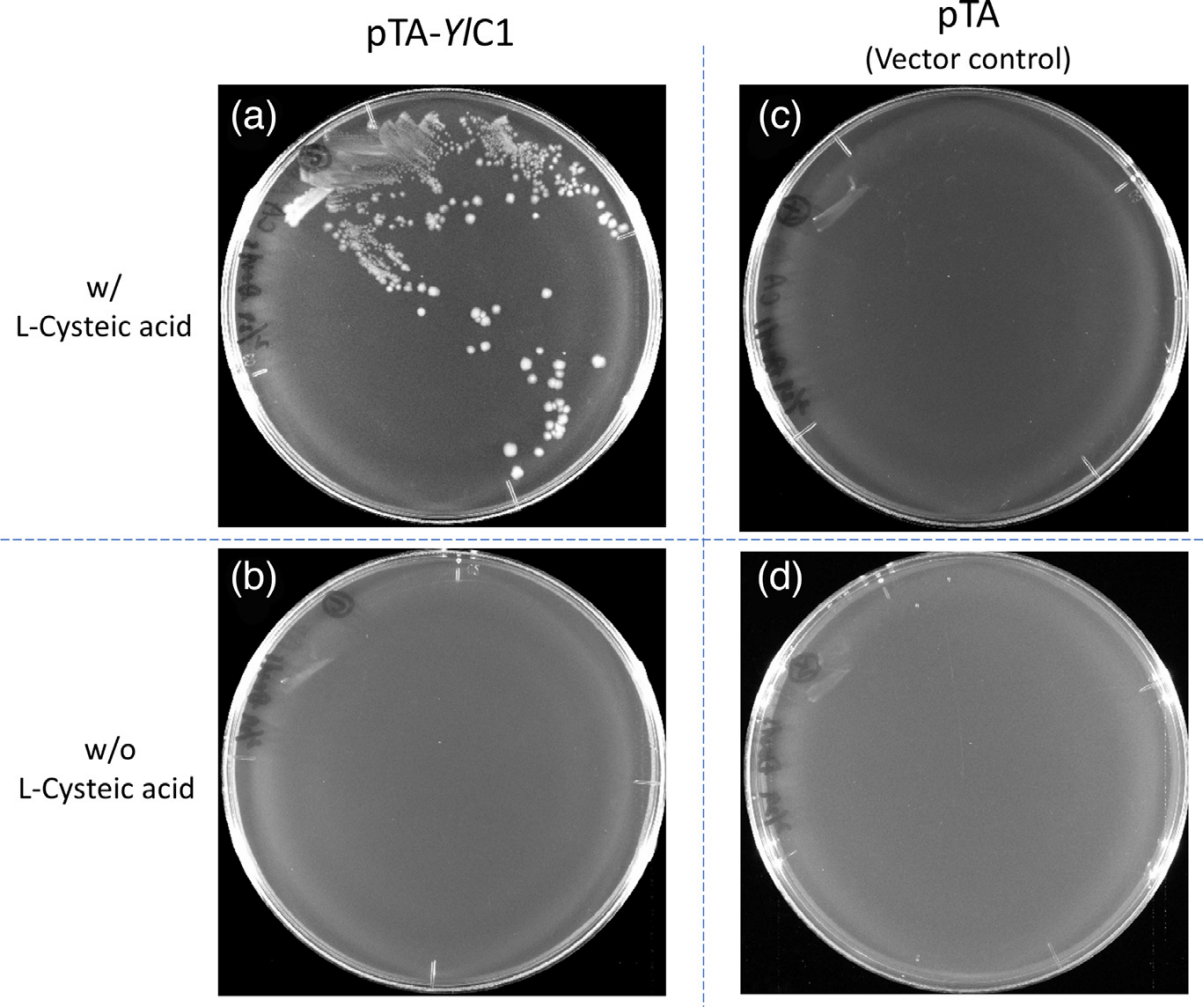
Identification of the *Y. lipolytica* CSAD gene using *E. coli* with optimized genetic mutations (*ΔcysA*, *ΔssuD* and *ΔcysC*). The *E. coli* strain CR2SC, harbouring the pTA-*Yl*C1 plasmid, was streaked onto Davis minimal agar plates with or without 0.5 mM l-cysteic acid. Both plates contained ampicillin to ensure plasmid maintenance. The cells were incubated at 37 °C for 3 days. As a control, CR2SC harbouring the empty vector pTA was cultured under the same conditions to confirm the specificity of the observed activity.

A gene encoding an aa sequence identical to that of the protein YALI0C16753p was chemically synthesized and placed downstream of the *E. coli trpL* promoter on the plasmid pTA to generate pTA-*Yl*C1 for efficient expression in *E. coli*. As shown in Fig. 3, CR2SC (*ΔcysA ΔssuD ΔcysC*) harbouring pTA-*Yl*C1 grew on Davis minimal agar containing 0.5 mM l-cysteic acid (a) but did not grow in its absence (b). CR2SC harbouring pTA (empty vector) did not grow well, regardless of l-cysteic acid presence (c, d). This strongly suggests that the YALI0C16753p protein functions as CSAD in addition to GAD.

**Fig. 4.**
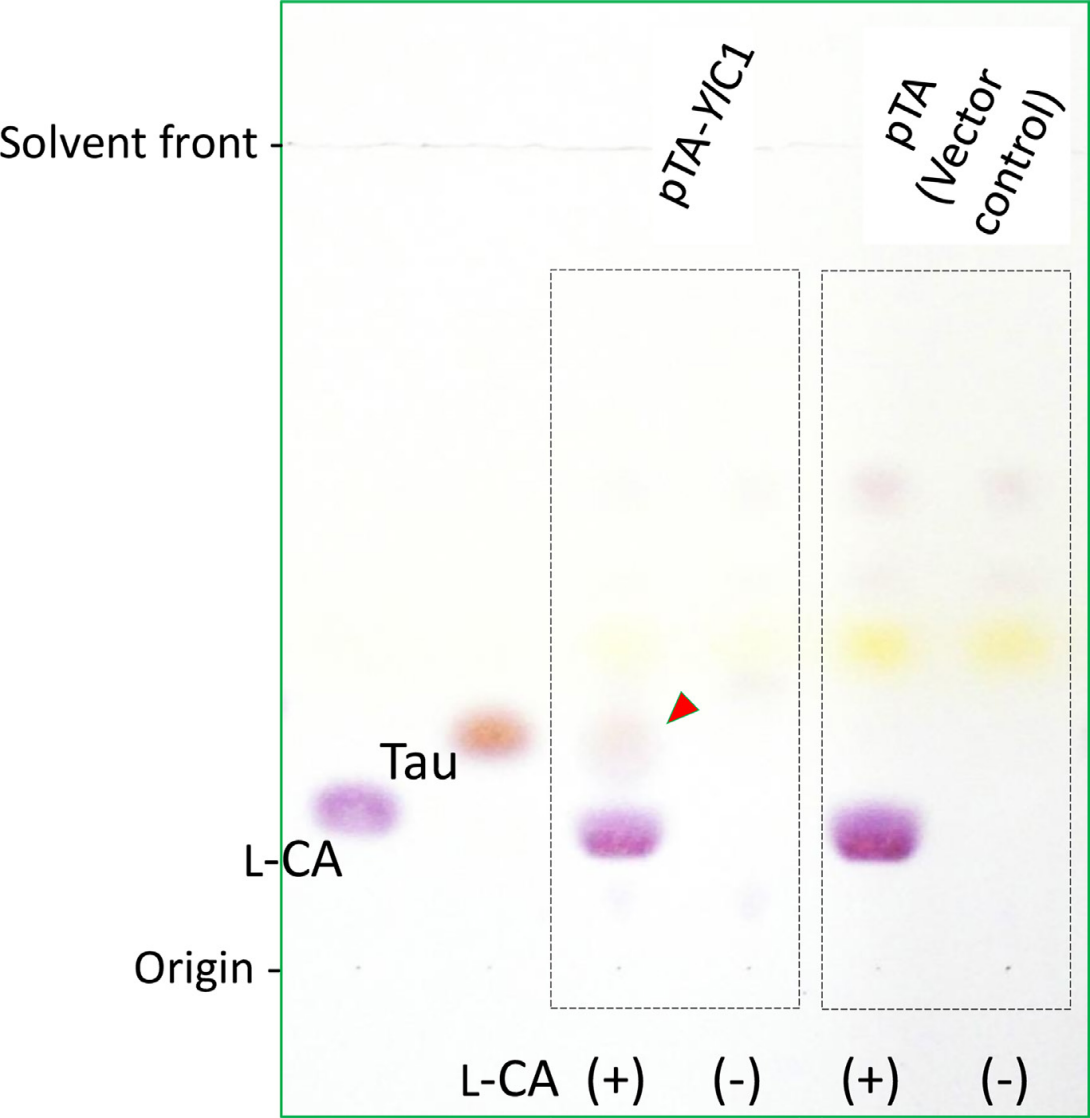
Validation of a candidate CASD gene for taurine synthesis via resting cell reaction and TLC analysis. Resting cell reactions were performed using the *E. coli* strain CR2SC carrying either the pTA-*Yl*C1 plasmid or the empty vector pTA. Following cell removal by centrifugation, the supernatant was analysed via TLC alongside 2 µg each of authentic l-cysteic acid (**l-**CA) and taurine (Tau). The labels L-CA(+) and L-CA(-) indicate the presence or absence of l-cysteic acid during the reaction. The arrowhead indicates a compound with properties consistent with taurine.

**Fig. 5.**
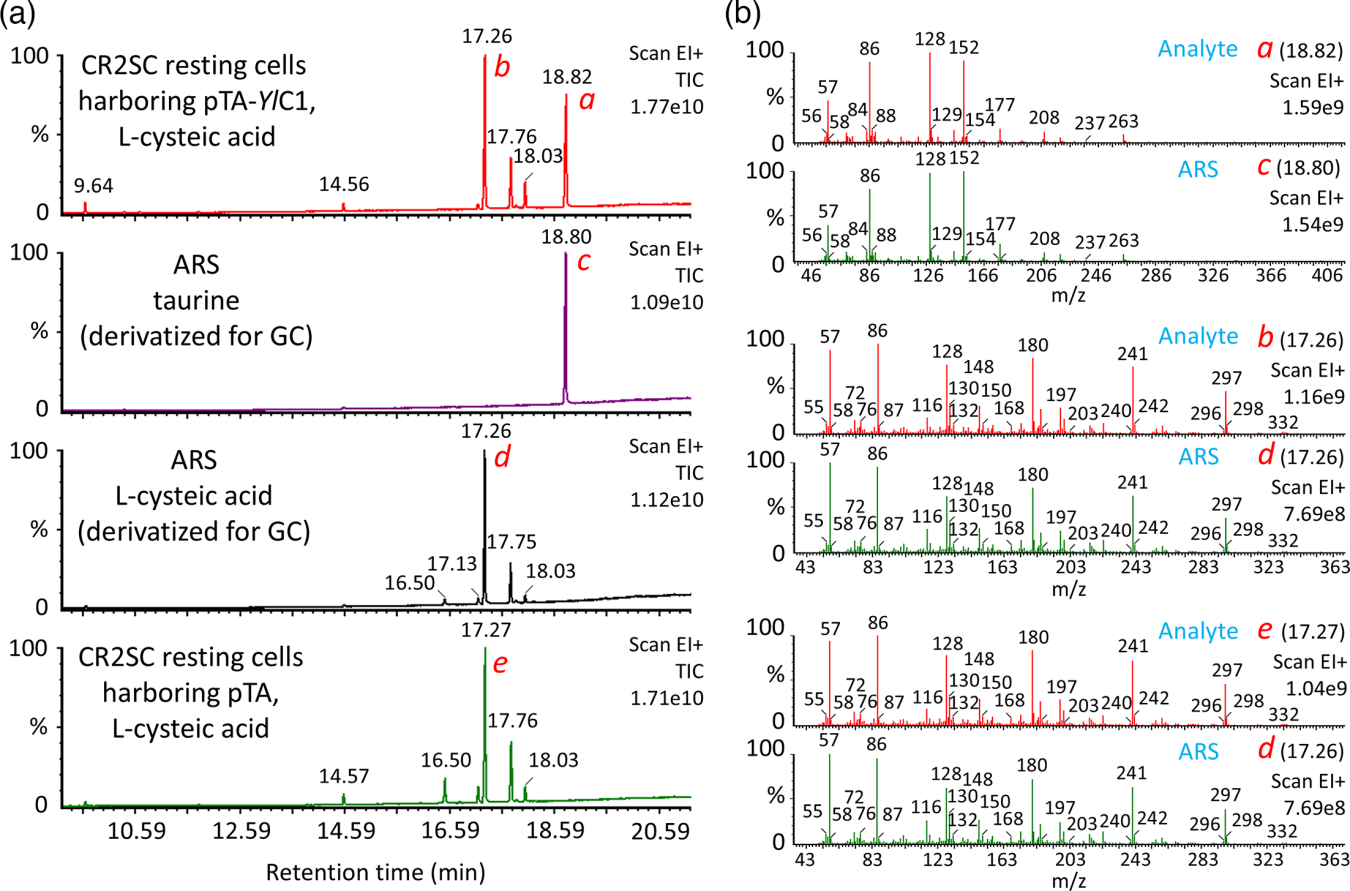
GC-MS analysis of reaction products from resting *E. coli* CR2SC cells harbouring a putative *Y. lipolytica* CSAD gene. Resting cell reactions were conducted with *E. coli* strain CR2SC harbouring either pTA-*Yl*C1 or the empty vector pTA. Supernatants from identical samples used for TLC (Fig. 4) were chemically derivatized to make them volatile for GC-MS analysis. ARS, taurine and l-cysteic acid were derivatized similarly. (**a**) Total ion current chromatogram (TIC). (**b**) Mass spectrum of peaks labelled *a–e* in panel (a). Retention times of the analysed peaks are shown in parentheses. The vertical scales (intensity) for TIC and mass spectra are relative, with the maximum peak height normalized to 100%. Peak height values are indicated in the upper right corners, such as 1.77e10. In panel (b), the signal at m/z 152, characteristic of taurine, was used to identify taurine in subsequent analyses (Fig. 6).

Next, the bioconversion of l-cysteic acid to taurine was confirmed using resting cells of CR2SC harbouring pTA-*Yl*C1. TLC detected a ninhydrin-positive taurine-like compound in the resting cell reaction only when the cells contained pTA-*Yl*C1 and l-cysteic acid was available (Fig. 4). In control experiments, no taurine-like compounds were observed. A more rigorous qualitative analysis was performed using GC-MS (Fig. 5). Taurine production during the resting cell reaction was demonstrated based on two observations: the retention time in GC separation matched that of the authentic reference standard (ARS), and the mass fragmentation pattern also matched that of ARS. It was concluded that the gene encoding YALI0C16753p is a genuine CSAD gene in *Y. lipolytica*.

**Fig. 6.**
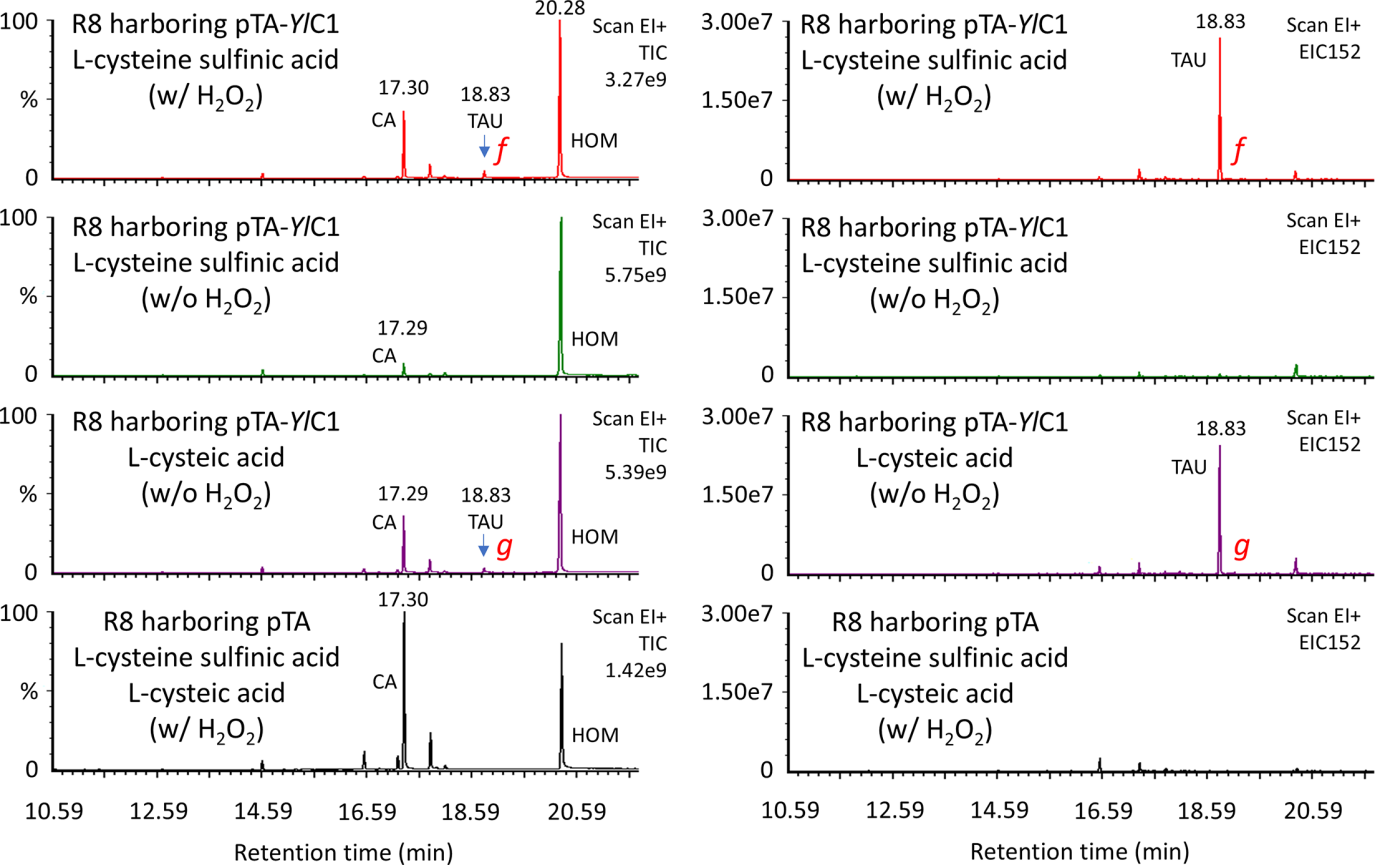
GC-MS analysis of enzymatic reaction products using crude cell-free extract of *E. coli* R8 containing a putative *Y. lipolytica* CSAD gene. Samples were derivatized for GC-MS analysis after the addition of 10 μmoles of homotaurine as an internal standard. Portions of the derivatized samples were injected into GC-MS, and the reaction substrates, including l-cysteine sulfinic acid, l-cysteic acid or their combination, are indicated on each chromatogram. Labels ‘w/ H_2_O_2_’ and ‘w/o H_2_O_2_’ indicate whether hydrogen peroxide was used to oxidize the sulfinic group to the sulfonic group before derivatization. The left column shows total ion current chromatograms (TIC). The vertical scales (intensity) are relative, with the TIC maximum peak height normalized to 100%. Maximum peak heights are shown in the upper right corners, such as 3.27e9. The right column displays extracted ion chromatograms (EIC) of m/z 152, the ion fragment characteristic of taurine (see Fig. 5b). The vertical scales (intensity) are absolute. TAU, taurine; CA, l-cysteic acid; HOM, homotaurine (internal standard).

Typically, CSAD is named for its activity in decarboxylating l-cysteine sulfinic acid. To examine whether the enzyme identified as CSAD, based on its activity in decarboxylating l-cysteic acid, also decarboxylates l-cysteine sulfinic acid, a crude enzyme solution prepared from *E. coli* R8 harbouring pTA-*Yl*C1 was tested. The sulfonic acid-specific derivatization for GC developed by Kataoka *et al*. [26] is not applicable to sulfinic acids. However, hypotaurine (a sulfinic acid) can be analysed by GC after conversion to taurine (a sulfonic acid) via hydrogen peroxide treatment prior to derivatization. As demonstrated in Figs 6, S4 and S5, the production of hypotaurine through the decarboxylation of l-cysteine sulfinic acid was indirectly confirmed by comparing the production of taurine with and without hydrogen peroxide treatment.

## Discussion

In this study, the CSAD gene from *Y. lipolytica* was successfully identified using recombinant DNA technology, incorporating an optimized combination of mutations in *E. coli*. Final confirmation of taurine production by the enzyme encoded by the *Y. lipolytica* gene of interest was achieved using l-cysteic acid as a substrate and analysing the results via GC-MS, a highly sensitive and precise analytical method. Furthermore, indirect evidence that the same enzyme utilizes l-cysteine sulfinic acid as a substrate to produce hypotaurine was obtained by comparing GC-MS results with and without hydrogen peroxide treatment of the products. Although signal heights for equimolar amounts of l-cysteic acid and hydrogen peroxide-treated l-cysteine sulfinic acid should ideally be identical, this was not observed in this study (Fig. S4). This discrepancy may be due to impurities, partial dissipation of hydration water in the reagents affecting accurate weighing or quantification uncertainties stemming from the omission of methylation of free carboxyl groups, as suggested by Kataoka *et al*. [26]. Nevertheless, the qualitative detection of taurine remained unaffected, as the conclusions were not compromised in any case (Fig. 6).

This method is extendable to the cloning of CSAD genes in other organisms. Two options are available depending on the expected likelihood of the CSAD gene being present in the sample population. If the target gene is to be identified from a sample already narrowed down based on genetic information, such as predictions of PLP-dependent enzymes acting on amino acids, a host lacking *cysA* and *ssuD* can be used, applicable in cases of relatively high probability. Conversely, if the target gene is to be identified from a broader sample, such as a whole genome library of a microorganism or a metagenomic library from soil, a host lacking one of *cysC*, *cysD* (sulfate adenylyltransferase subunit 2) or *cysN* in addition to *cysA* and *ssuD* is recommended, suitable for lower probability cases. In the former scenario, false positives, such as ‘gain-of-function’ mutations in *mscK,* are less likely to hinder CSAD gene detection. In contrast, in the latter scenario, introducing deletions like *cysC* helps reduce such false positives. However, hosts incapable of converting sulfate to sulfite, such as those lacking *cysC*, are disadvantaged, as they cannot produce l-cysteine, a key intermediate in taurine synthesis, from inexpensive sulfate – a drawback for practical applications. The loss of sulfate assimilation can impede experiments aiming to explore overall metabolism. When CysUWA is functional, trace external sulfate entering the cell can undesirably inhibit *tauD* expression [20]. To create a host with nonfunctional CysUWA, a *cysA*-deficient strain is suggested, which can be readily obtained using chromate resistance as an indicator. If the limitation caused by the loss of CysUWA function is undesirable, it may be circumvented by culturing a strain with the *mscK* ‘gain-of-function’ mutation allele identified in this study in a medium with high sulfate concentration. Thus, in addition to optimizing the combination of mutations, designing the medium composition is a critical factor influencing the sensitivity of CSAD gene detection. Considering the screening procedure, there is a possibility that SsuD homologues capable of desulfonating l-cysteic acid could be mistaken for CSADs. Therefore, definitive validation using simple methods, such as TLC, is recommended, particularly in cases with a low probability of CSAD gene presence.

Two new insights were serendipitously uncovered during the development of the screening system. First, deleting *ssuD* eliminated background growth reliant on an unidentified sulfur source. While it is known that SquD and SmoC, homologues of SsuD in *Novosphingobium aromaticivorans* and *Agrobacterium tumefaciens*, respectively, can cleave the C–S bond in sulfoquinovose [37,38], the presence of sulfoquinovose in the culture plate agar remains uncertain. The unidentified sulfonic acid compounds derived from agar, including but not limited to sulfoquinovose, may serve as substrates for SsuDE activity in *E. coli* and warrant further investigation. Second, during the cultivation of *E. coli* strain CR2S (*cysA ssuD*) on Davis minimal agar containing l-cysteic acid, mutants with *mscK* mutations were easily selected. For instance, a mutant harbouring *mscK1* (Leu791Pro) acquired the ability to grow on Davis minimal agar without l-cysteic acid. Deleting *mscK1* in this mutant caused it to revert to the WT phenotype, resulting in a loss of its ability to grow on the same agar and confirming that the mutant exhibits a ‘gain of function’. Previous studies have shown that channels formed by mutant proteins with an Arg792Pro substitution display abnormal gating [39]. This study suggests that extracellular sulfate enters CR2S-M1 cells via the channel formed by MscK1 with the Leu791Pro substitution, a residue adjacent to the one identified in the earlier report. The extracellular sulfate concentration required for MscK1-mediated sulfur influx is significantly higher than that required by the CysUWA transporter. Furthermore, sulfate entry through MscK1 channels is inhibited by ~204 mM Na^+^. However, under these restrictive conditions, new mutants can emerge, enabling the cells to overcome sulfur starvation. For example, in one such mutant, strain CR2S-M1-2, which has a short deletion in *clsA* (cardiolipin synthase A), MscK1-mediated sulfate-sulfur assimilation was not inhibited at 204 mM Na^+^ and 22 mM K^+^. This observation that sodium ion affects mutant-type potassium-dependent MscK channels could offer previously unexplored insights into MscK function [39].

There are likely several reasons why *mscK* mutants compensating for the sulfate transporter CysUWA deficiency were isolated in this study using Davis minimal medium. In addition to the effect of l-cysteic acid discussed below, this may be due to the relatively high sulfate ion concentrations and low sodium ion concentrations in the Davis minimal agar. This emphasizes the need to carefully consider the composition of minimal media to minimize false positives.

l-Cysteic acid in Davis minimal medium promotes the emergence of *mscK* ‘gain-of-function’ mutants with enhanced sulfate uptake within 4 days of incubation, although, the reason remains uncertain (Fig. 1e). Similar mutants also appear after prolonged incubation, even without l-cysteic acid (Fig. 1d). To the best of my knowledge, there are no reports suggesting that l-cysteic acid is mutagenic. Future studies should investigate whether l-cysteic acid influences *mscK* expression, specifically if its presence increases *mscK* expression compared with its absence, potentially favouring the selection of mutant *mscK* cells. Expression of *mscK* is reported to be negatively regulated by the transcription factor Lrp (leucine-responsive transcriptional regulator) [40]. The transcriptional regulation of *cysB*, the master regulator of cysteine biosynthesis, depends on multiple factors, one of which regulates *cysB* similarly to how Lrp regulates *mscK*. However, understanding how l-cysteic acid drives the emergence of *mscK* mutants is beyond the scope of this study and remains a hypothesis for future exploration.

Currently, the method developed in this study is limited to identifying whether the gene of interest or a gene selected from a pool of miscellaneous genes is a CSAD. The next step is to refine this method for evaluating CSAD variants or mutants that improve taurine synthesis efficiency. One potential approach is to utilize the properties of taurine and hypotaurine as compatible solutes that enhance salt tolerance and as antioxidants that protect cells against oxidative stress inducers such as menadione [41,44], allowing for better screening methods.

## Supplementary material

10.1099/mic.0.001620 Uncited Supplementary Material 1.

## Abbreviations

ARS: authentic reference standard
CDO: cysteine dioxygenase
CSAD: cysteine sulfinic acid decarboxylase
GAD: glutamic acid decarboxylase
LB: Luria-Bertani
