# Supplementary material for: Identification of the Yarrowia lipolytica cysteine sulfinic acid decarboxylase gene using a newly developed method with optimized Escherichia coli combinations of mutant alleles
Source: Microbiology (Reading). 2025 Nov 4;171(11):001620. doi: 10.1099/mic.0.001620 (PMC12585060; doi:10.1099/mic.0.001620)
Supplement: Uncited Supplementary Material 1. [file mic-171-01620-s001.pdf]

Hypothetical biosynthetic pathway of taurine derived from L-cysteine

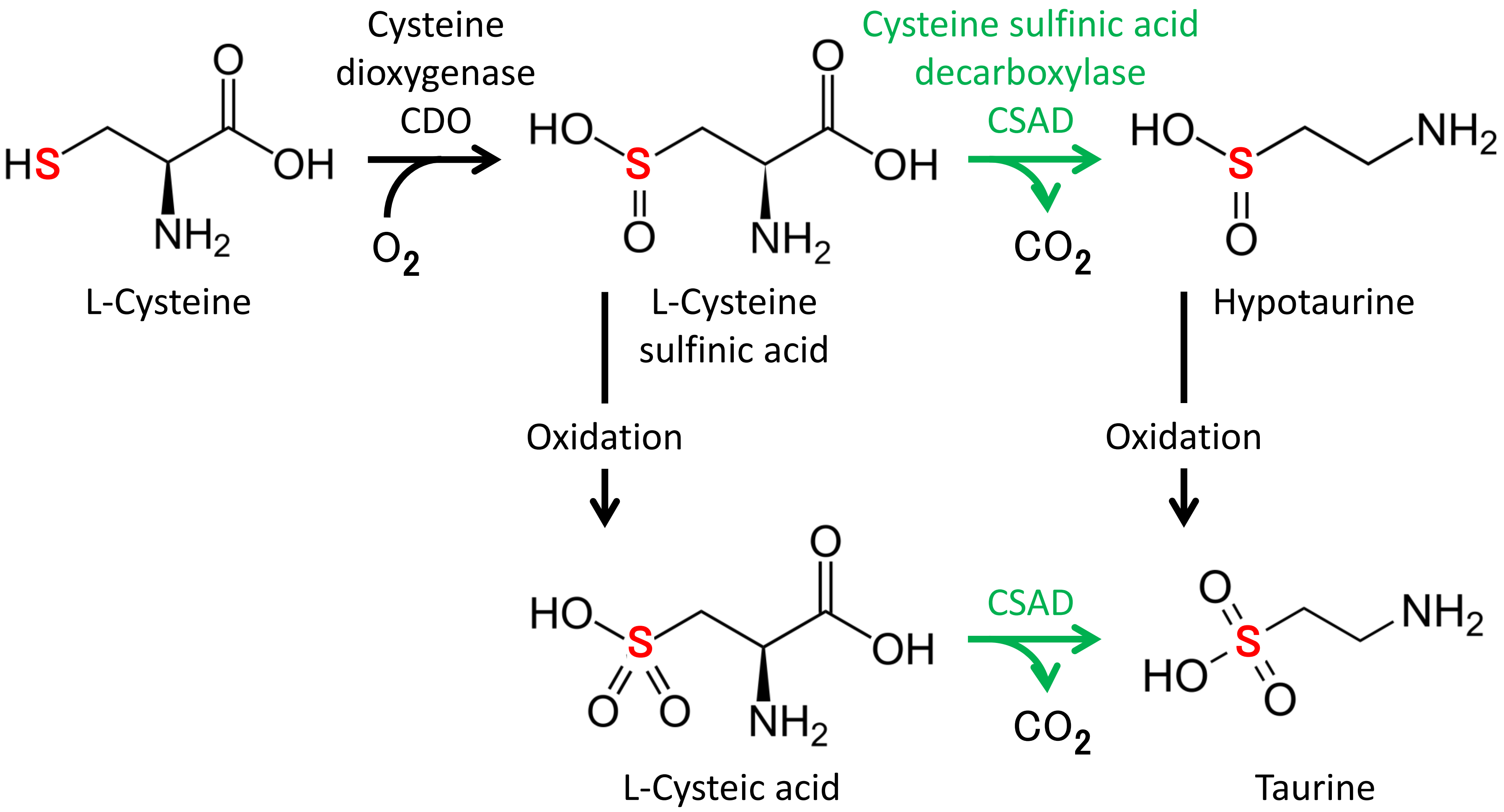

Fig. S1

A sensitive method was developed for screening the cysteine sulfinic acid decarboxylase (CSAD) gene, utilizing the L-cysteic acid-dependent growth of an *Escherichia coli* host with optimized genetic backgrounds.

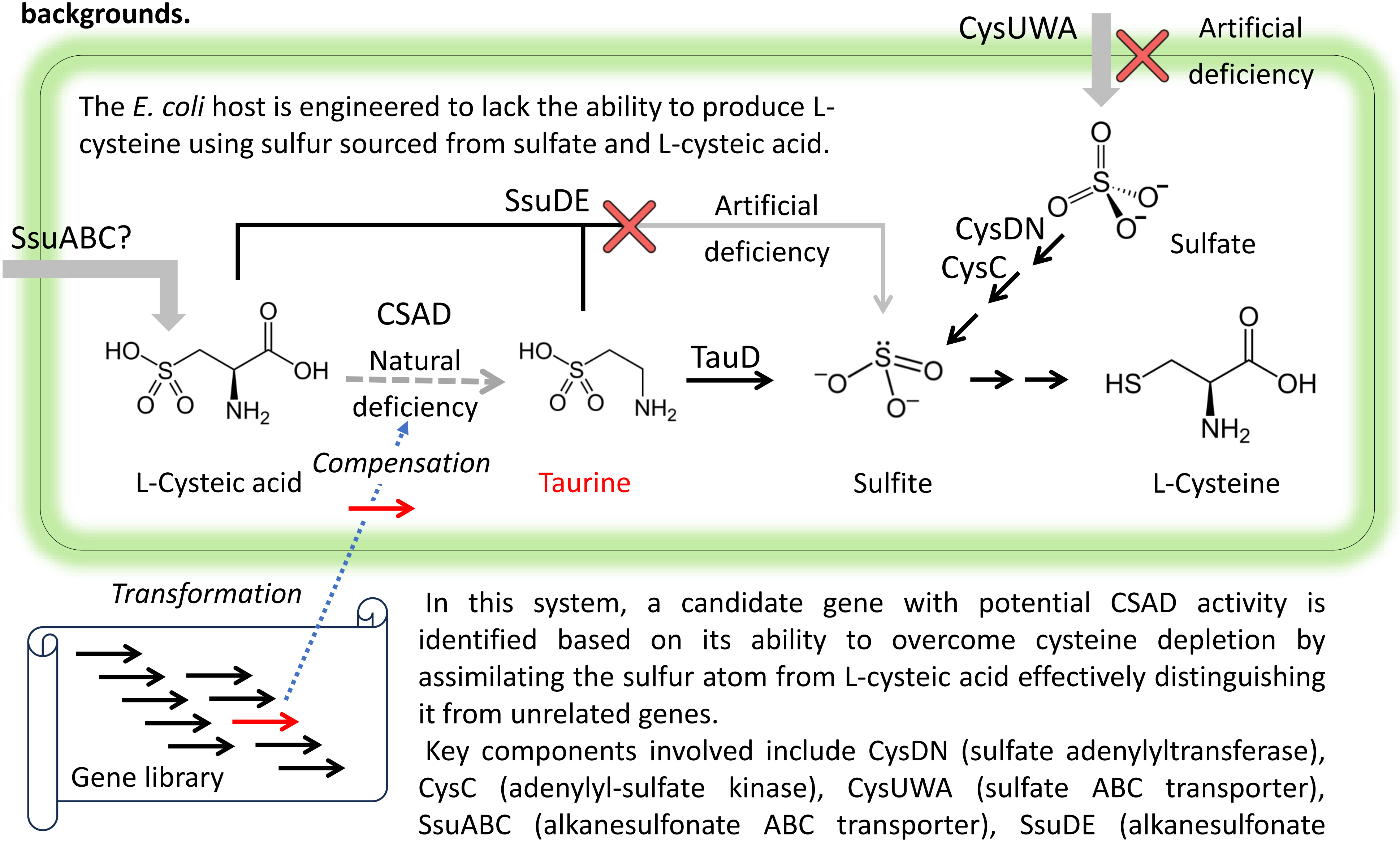

In this system, a candidate gene with potential CSAD activity is identified based on its ability to overcome cysteine depletion by assimilating the sulfur atom from L-cysteic acid effectively distinguishing it from unrelated genes.

Key components involved include CysDN (sulfate adenylyltransferase), CysC (adenylyl-sulfate kinase), CysUWA (sulfate ABC transporter), SsuABC (alkanesulfonate ABC transporter), SsuDE (alkanesulfonate monooxygenase/NADPH-dependent FMN reductase), and TauD (taurine dioxygenase).

Fig. S2

Sulfate uptake by a "gain-of-function" MscK mutant requiring high extracellular sulfate concentrations and its inhibition by NaCl

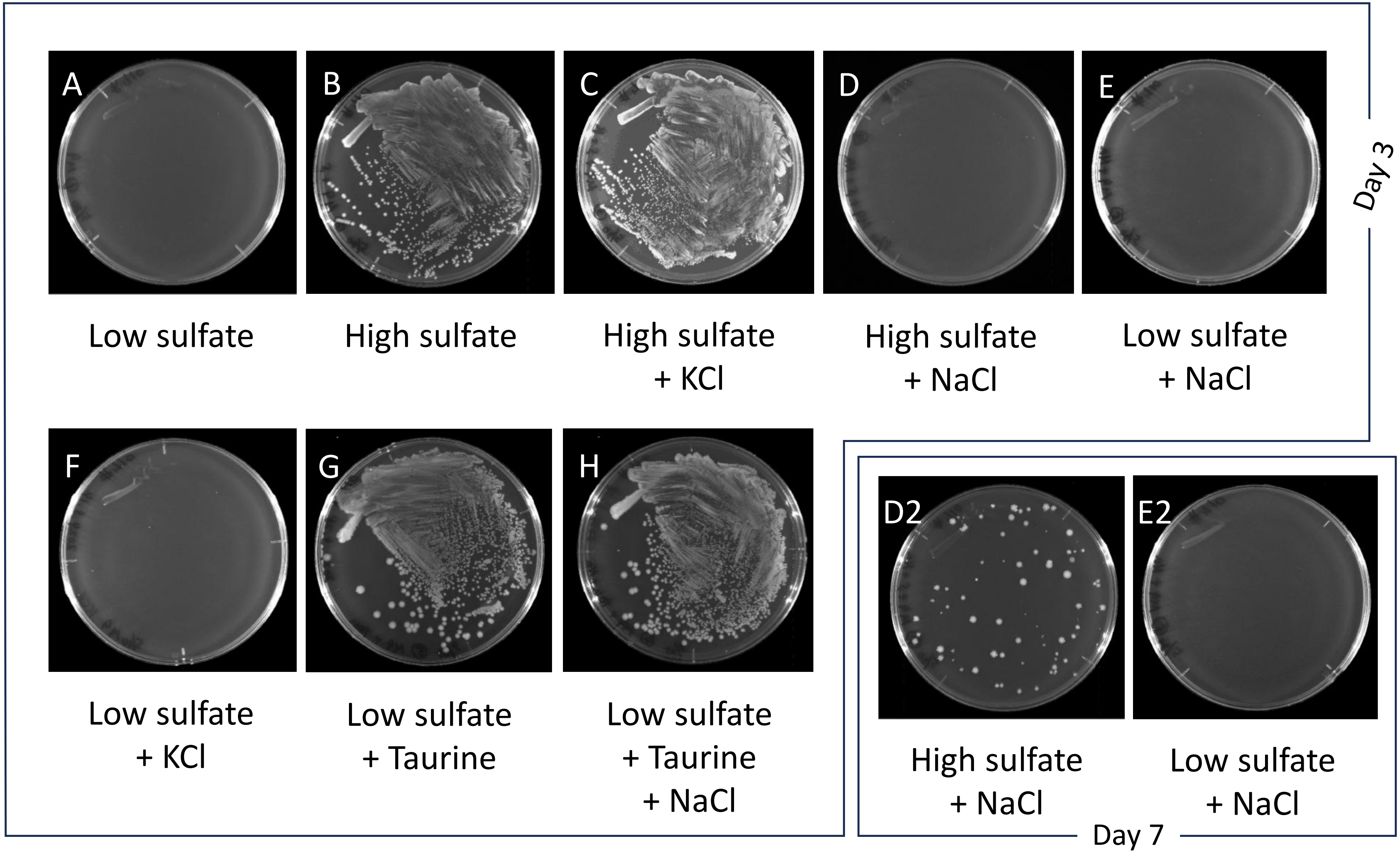

Fig. S3 (continued below)

The *Escherichia* strain CR2S-M1 (*mscK1*) was cultured on M9 minimal agar plates at 37° C for three to seven days. M9 minimal medium is relatively high in Na^+ and low in K^+, whereas Davis minimal medium is relatively low in Na^+ and high in K^+. "Low sulfate" indicates a sulfate concentration of ~2.8 mM derived from agar, while "high sulfate" reflects the addition of ammonium sulfate to reach a total sulfate concentration of ~11.2 mM, equivalent to that in Davis minimal agar medium. Labels "+ KCl", "+ NaCl", and "+ Taurine" indicate the addition of potassium chloride, sodium chloride, or taurine to achieve final concentrations of ~122 mM K^+, ~204 mM Na^+, and 1 mM taurine, respectively. In the absence of these additions, the medium contained 22 mM K^+ and 104 mM Na^+.

Fig. S3

Gas chromatography-mass spectrometry (GC-MS) analysis of authentic reference standards

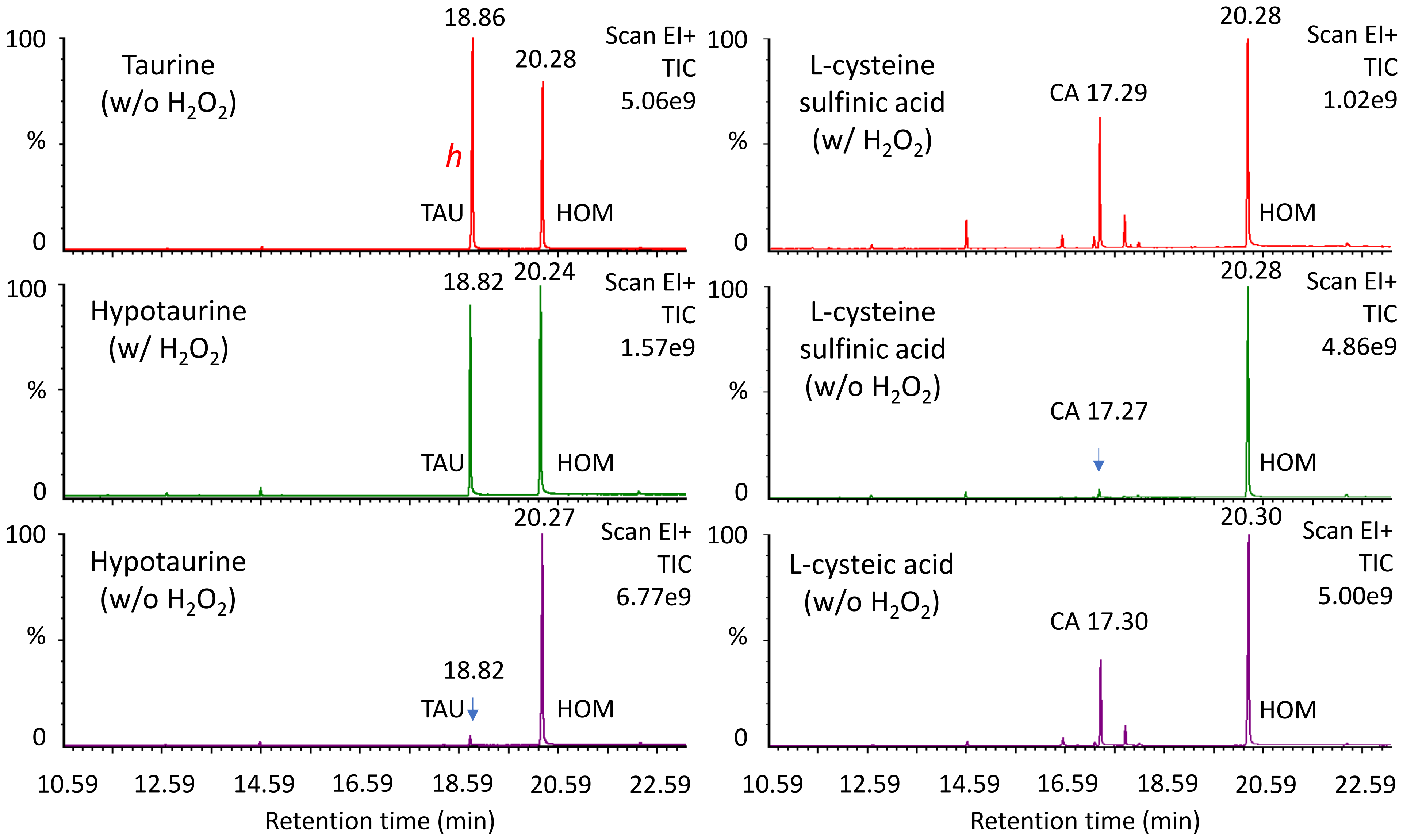

Fig. S4 (continued below)

A total of 10 μmoles each of taurine, hypotaurine, L-cysteine sulfinic acid, and L-cysteic acid were derivatized for GC-MS analysis with 10 μmoles of homotaurine as an internal standard. Portions of the derivatized samples were injected into the GC-MS. Chromatograms labeled "w/ H_2O_2" and "w/o H_2O_2" indicate whether hydrogen peroxide was used to oxidize the sulfinic group to the sulfonic group before derivatization. The vertical scales (intensity) of the total ion current chromatograms (TIC) are relative, with the maximum peak height normalized to 100%. Maximum peak heights are shown in the upper right corners, such as 5.06e9. Arrows highlight minor signals corresponding to taurine and L-cysteic acid, produced via the autooxidation of hypotaurine and L-cysteine sulfinic acid, respectively. Abbreviations: TAU, taurine; CA, L-cysteic acid; HOM, homotaurine (internal standard).

Fig. S4

Gas chromatography-mass spectrometry (GC-MS) analysis of enzymatic reaction products using crude cell-free extract of *Escherichia coli* R8 containing a putative *Yarrowia lipolytica* CSAD gene

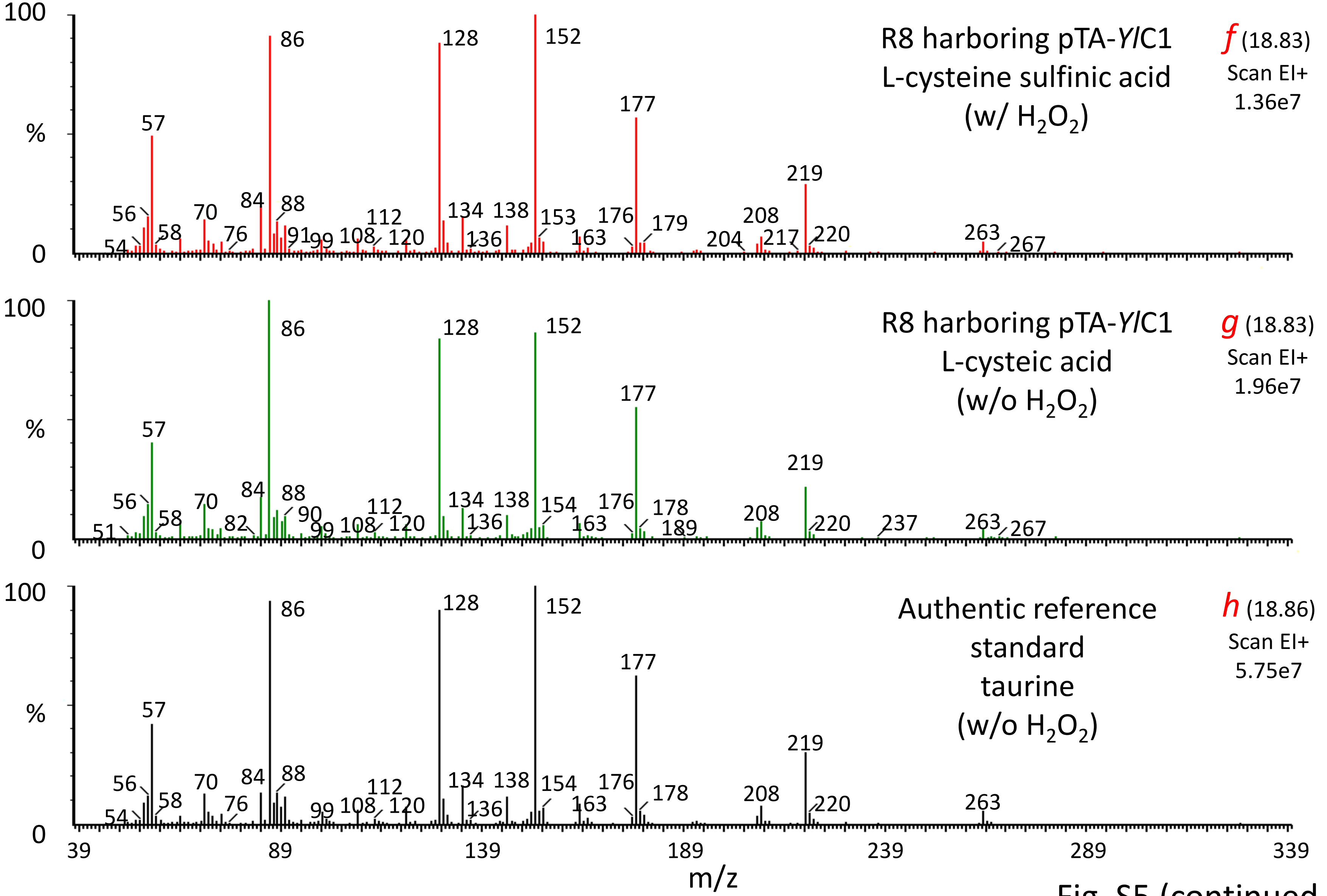

Fig. S5 (continued below)

This figure presents the mass spectrum of peaks labeled *f*, *g* (from Fig. 6) and *h* (from Fig. S4). Retention times of the analyzed peaks are shown in parentheses. The vertical scales (intensity) are relative, with the maximum peak height normalized to 100%. Maximum peak heights are indicated in the upper right corners, such as 1.36e7.

Fig. S5
